# A scoping review of advancements in machine learning for glaucoma: current trends and future direction

**DOI:** 10.3389/fmed.2025.1573329

**Published:** 2025-04-24

**Authors:** Jiatong Zhang, Bocheng Tian, Mingke Tian, Xinxin Si, Jiani Li, Ting Fan

**Affiliations:** ^1^The First Clinical Medical School, China Medical University, Shenyang, China; ^2^The Second Clinical Medical School, China Medical University, Shenyang, China; ^3^Emory College of Arts and Sciences, Emory University, Atlanta, GA, United States; ^4^The Fourth Clinical Medical School, China Medical University, Shenyang, China; ^5^School of Intelligent Medicine, China Medical University, Shenyang, China

**Keywords:** machine learning, glaucoma diagnosis, deep learning, multimodal imaging, ophthalmic research

## Abstract

**Introduction:**

Machine learning technology has demonstrated significant potential in glaucoma research, particularly in early diagnosis, predicting disease progression, evaluating treatment responses, and developing personalized treatment strategies. The application of machine learning not only enhances the understanding of the pathological mechanism of glaucoma and optimizes the diagnostic process but also provides patients with accurate medical services.

**Methods:**

This study aimed to describe the current state of research, highlight directions for further development, and identify potential trends for improvement. This review was conducted following the scoping review of the Preferred Reporting Items for Systematic Reviews and Meta-Analyses (PRISMA) extension to showcase advancements in the application of machine learning in glaucoma research and treatment.

**Results:**

We employed a comprehensive search strategy to retrieve literature from the Web of Science Core Collection database, ultimately including 3,581 articles in the analysis. Through data analysis, we identified current research hotspots, noted differences in researchers' attitudes and opinions, and predicted potential future development trends.

**Discussion:**

We divided the research topics into six categories, clearly identifying “eye diseases”, “retinal fundus imaging” and “risk factors” as the key terms for the development of this field. These findings signify the promising prospects of machine learning, particularly when integrated with multimodal technologies and large language models, to enhance the diagnosis and treatment of glaucoma.

## 1 Introduction

Glaucoma, characterized by retinal nerve fiber layer defects and concomitant visual field (VF) damage, is considered one of the leading causes of irreversible visual impairment worldwide (1). Glaucoma is estimated to affect more than 110 million individuals over the next two decades (2, 3). Therefore, adopting novel diagnostic techniques to explore glaucoma pathogenesis and facilitate early detection protocols is of significant clinical relevance (4, 5).

In 2016, a significant shift occurred in the application of machine learning in the realm of ophthalmology with the publication of articles focusing on its applications in diabetic retinopathy (DR) screening (6, 7). Subsequently, the integration of medical imaging with machine learning technology has been applied to disease diagnosis, and researchers have developed numerous automated ophthalmic diagnostic systems (8–11). Deep learning (DL) algorithms, which extract features from images and represent them in layers, have shown considerable promise in distinguishing between glaucomatous and non-glaucomatous patterns (12, 13). Moreover, the DL model performed well in the fundus image analysis of glaucoma, with a diagnostic accuracy of 95.59% (14). These algorithms are critical for screening, diagnostic decision-making, and disease prognosis (15). The emergence of machine learning-based multimodal imaging technologies is poised to have a transformative impact on medical management and therapeutic strategies for glaucoma in the coming years (16).

In recent years, the growing body of literature on machine learning applications in glaucoma research has attracted significant scholarly interest, reflecting the dynamic evolution of the field and the imperative for systematic evaluation. During this period, a comprehensive assessment and review of existing knowledge can be performed using methods such as statistical analysis of publication volume, collaboration across countries, regions, or institutions, analysis of the sources and characteristics of cited publications, and identification of high-frequency and emerging keywords. These approaches can clarify the current research hotspots in advancing glaucoma diagnosis and treatment with machine learning and predict future development directions. Currently, advancements in machine learning within the field of glaucoma are mostly fragmented, with a lack of comprehensive and forward-looking reviews. Therefore, we conducted an assessment and scoping review of relevant articles to assess and summarize the existing knowledge about the application of machine learning in glaucoma research. We aimed to clarify the role of machine learning in advancing glaucoma research and identify key research directions through a visual approach, considering the development trends in this field (17, 18). Our report may provide some references for ophthalmologists and medical engineering researchers to comprehensively explore this field and embrace machine learning applications in glaucoma.

## 2 Methods

### 2.1 Scoping review

For this scoping review, we selected the Web of Science Core Collection database, as it offers comprehensive subject coverage and high-quality literature, providing a wide range of influential, representative, and accurate literature on the research topic. We developed a search strategy that covers a wide range of themes, which, unlike systematic reviews, does not rely solely on the quality of evidence as the only measure. This approach allows for better acquisition of researchers' perspectives and attitudes within the broad scope of the study. We also extracted and identified multiple characteristics of the retrieved literature, including country, institution, references, journals, subject categories, and keywords. This enables our research to gain a more comprehensive understanding of the development status within the field, thereby making more accurate predictions about development trends.

### 2.2 Data retrieval

We developed an accurate and comprehensive search query using the following search terms: (TS= ((“Machine Learning” OR “Artificial Intelligence” OR “Deep Learning” OR “Support Vector Machine^*^” OR “Linear Regression” OR “Logistic Regression” OR “decision tree” OR “random forest” OR “K-Nearest Neighbors” OR” Naive Bayes” OR “Naive Bayes Model” OR “Convolutional Neural Network^*^” OR “Recurrent Neural Network^*^” OR “XGBoost” OR “Fully Convolutional Network^*^” OR “Generative Adversarial Network” OR “Reinforcement Learning” OR “Back Propagation” OR “Fully Neural Network” OR “Recursive Neural Network” OR “Auto encoder” OR “Deep Belief Network” OR “Restricted Boltzmann machine” OR “Transformers” OR “Graph Convolution Networks” OR “k-means” OR “Ada boost” OR “Markov chain” OR “Natural Language Processing” OR “Generative Pre-trained Transformer” OR “Bidirectional Encoder Representations from Transformers” OR “LLM”) AND (“Glaucoma”))), the literature type= “Article”. A literature search was conducted from 2000 to 2024.

### 2.3 Inclusion and exclusion verification

In terms of publication time, to study the latest advancements in machine learning within the field of glaucoma, we focused on relatively recent literature, excluding studies published before 2000. Regarding article types, we selected “articles” to ascertain the perspectives and attitudes of relevant researchers, as these formats are less influenced by external opinions during the writing process and can clearly express personal viewpoints. We excluded evidence types such as “conferences”, “article reviews”, “letters”, and “news”. For search topics, we extracted keywords from the research questions and, based on these, obtained clusters of related words, which were used as search terms to ensure a comprehensive and objective search of the topic keywords. To ensure that the articles found aligned with our research theme, two researchers independently verified the retrieved data.

### 2.4 Data feature recognition

We extracted various metadata from the retrieved publications, including article titles, abstracts, authorship, institution, country/region, journal, keywords, and cited references, to create a connected network of 3,581 articles with 80,657 references.

### 2.5 Data classification analysis

Data analysis was performed from multiple perspectives, including publication year, country/region, institution, journal, cited literature, high-frequency keywords, and emerging keywords. This approach allowed us to study the characteristics of researchers in terms of changes in word preference, selection of publication journals, and shifts in word frequency, thereby identifying current research hotspots and variations in researchers' attitudes and viewpoints (19).

### 2.6 Summary and visualization presentation

We converted the data into easily recognizable charts or images and conducted predictive research on the development direction of researchers' views and attitudes toward the subject. Based on the knowledge obtained from data classification analysis, we predicted future trends and potential research directions in this field.

### 2.7 Patient consent

This study was a scoping review, and the clinical data used were derived from public databases; therefore, informed consent from patients could not be obtained.

## 3 Results

### 3.1 Distribution of articles by publication years

**Figure 1:**
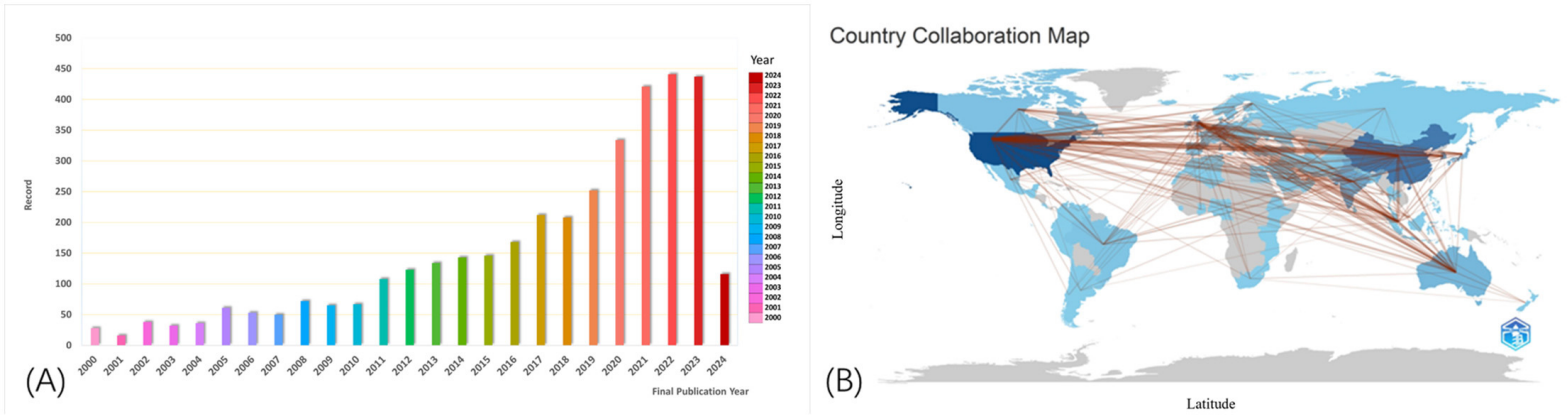
Numbers of published articles from 2000 to 2024 **(A)**. The cooperation of countries or regions that contributed to publications **(B)**. Collaboration between countries provides a strong incentive for progress in scientific studies. The lines represent the collaboration between different countries, with broader lines indicating more collaboration and communication.

We retrieved a total of 3,581 published articles. As shown in Figure 1A, the bars represent the total annual number of published studies, illustrating the publication trend from 2000 to 2024. Notably, the number of published articles has steadily increased, at a slow pace from 2000 to 2016, followed by a significant rise in recent years. Over the last 4 years, this number has accounted for more than 11% of the total number of published articles. These findings demonstrate that the boom in machine learning technology and its application in glaucoma is gradually becoming the focus of attention and entering a stage of rapid development. The number of publications in 2024 was significantly lower than in 2023. This is because the information we retrieved on 1 April 2024, only included literature published before that date and did not encompass publications released after 1 April 2024.

### 3.2 Analysis of countries/regions, institutions, and journals

We analyzed the literature within the reference range, which included publications from 102 countries and 3,343 institutes. The country that has contributed the most to the literature is the United States, followed by China, South Korea, England, and India, which rank second to fifth, respectively. Most of the influential research institutions are located in the United States and the United Kingdom, indicating that these countries and institutions have invested more research efforts in this field and should be given attention. However, it is worth mentioning that the research center with the highest number of published articles is the University of California system (300 articles), highlighting its prominent role and significant impact in the field of machine learning applications for glaucoma.

As shown in Figure 1B made by the bibliometrix package in R software shows how articles were distributed in the various contributing countries and areas. Collaboration between countries is a strong incentive for progress in scientific studies. The lines refer to the work between different countries. The broader lines indicate more collaboration and communication.

### 3.3 References analyses

References are key indicators, as frequently cited documents can significantly influence their research areas. Table 1 lists the top 10 most-cited articles in this field, which were cited more than 470 times out of 80,657 articles. The paper published in the Journal of the American Medical Association by Ting, DSW, Wong, TY, et al., which has been cited 1,369 times, ranks first (20). This article is the most frequently cited within this field of research.

**Table 1:** Top 10 most-cited references on machine learning for glaucoma applied.

**Rank**	**Title of the cited document**	**DOI**	**Times cited**	**Interpretation of findings**
1	Development and Validation of a Deep Learning System for Diabetic Retinopathy and Related Eye Diseases Using Retinal Images From Multiethnic Populations With Diabetes (20).	10.1001/jama.2017.18152	1,369	This study demonstrated that DL systems exhibit high true positive rates and true negative rates in detecting DR and related eye diseases among multiethnic populations with diabetes. However, further research is required to assess their applicability in healthcare settings and their effectiveness in improving visual outcomes.
2	Pivotal trial of an autonomous AI-based diagnostic system for detection of diabetic retinopathy in primary care offices (69).	10.1038/s41746-018-0040-6	793	This study marked the first Food and Drug Administration (FDA)-approved autonomous AI diagnostic system, which demonstrated high sensitivity and specificity in detecting DR, potentially helping thousands of diabetic patients prevent vision loss each year.
3	Joint Optic Disc and Cup Segmentation Based on Multi-Label Deep Network and Polar Transformation (70).	10.1109/TMI.2018.2791488	619	The multilayer network (M-Net), a DL architecture, effectively segments the optic disc and optic cup in fundus images through a one-stage multi-label system, improving the accuracy of glaucoma screening and diagnosis by calculating the cup-to-disc ratio.
4	Optical Coherence Tomography Angiography of Optic Disc Perfusion in Glaucoma (71).	10.1016/j.ophtha.2014.01.021	582	Optical coherence tomography (OCT) angiography, using the split-spectrum amplitude-decorrelation angiography algorithm, reliably measures optic disc perfusion and reveals reduced perfusion in patients with glaucoma, highlighting its potential as a tool for assessing glaucoma and monitoring its progression.
5	Predictive factors for glaucomatous visual field progression in the advanced glaucoma intervention study (72).	10.1016/j.ophtha.2004.02.017	561	The Advanced Glaucoma Intervention Study found that older age and greater intraocular pressure fluctuation are significant risk factors for VF progression in glaucoma, increasing the odds by 30% for each 5-year increase in age and 1-mmHg rise in intraocular pressure fluctuation, respectively.
6	Determinants of normal retinal nerve fiber layer thickness measured by stratus OCT (73).	10.1016/j.ophtha.2006.08.046	515	Retinal nerve fiber layer thickness, as measured by Stratus OCT, is significantly influenced by age, ethnicity, axial length, and optic disc area. These factors should be considered in the diagnosis and monitoring of glaucoma.
7	Optical Coherence Tomography Angiography of the Peripapillary Retina in Glaucoma (74).	10.1001/jamaophthalmol.2015.2225	514	OCT angiography can visualize and quantify reduced peripapillary retinal perfusion in glaucomatous eyes, which is strongly correlated with VF defects, highlighting its potential as a valuable tool for glaucoma evaluation.
8	Efficacy of a Deep Learning System for Detecting Glaucomatous Optic Neuropathy Based on Color Fundus Photographs (75)	10.1001/10.1016/j.ophtha.2018.01.023	495	This study demonstrates that the deep learning system achieved exceptional diagnostic accuracy (AUC 0.986) in detecting referable glaucoma-related optic nerve damage using fundus photographs, with high sensitivity (95.6%) and specificity (92%).
9	Prevalence of ocular surface disease in glaucoma patients (76).	10.1097/IJG.0b013e31815c5f4f	480	The study revealed a high prevalence of ocular surface disease in patients with glaucoma, with benzalkonium chloride-containing eye drops being significantly associated with increased odds of abnormal clinical test results.
10	Comparing Adherence and Persistence Across six Chronic Medication Classes (77).	10.18553/jmcp.2009.15.9.728	477	The study revealed suboptimal medication adherence across chronic therapies, with the lowest rates for prostaglandin eye drops and overactive bladder medications.

### 3.4 High-frequency keyword analyses

We conducted a systematic extraction of keywords from 3,581 articles, and the top 25 with highest occurrences are listed (Table 2). While identifying thematic areas in fields of investigation, a closer examination of these keywords used in the papers revealed that 25 keywords occurred at least 125 times.

**Table 2:** Top 25 occurrence keywords.

**Keywords**	**Count**	**Keywords**	**Count**
glaucoma	699	diagnosis	196
open-angle glaucoma	658	classification	191
prevalence	578	ocular hypertension	181
intraocular-pressure	398	damage	179
optical coherence tomography	352	risk	175
risk factors	352	normal-tension glaucoma	146
progression	322	images	145
eyes	320	optic disc	141
population	285	eye	131
nerve-fiber layer	255	age	128
thickness	241	visual field	128
association	201	segmentation	126
diabetic retinopathy	199		

**Figure 2:**
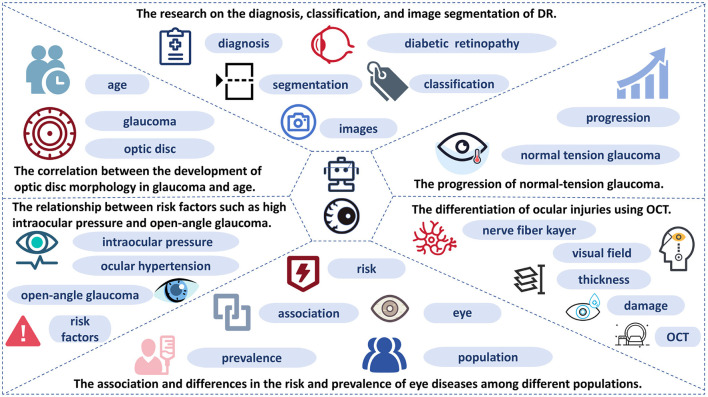
Dendrogram shows the thematic areas in research of machine learning in glaucoma.

We used the VOSviewer to analyze the relationships between high-frequency keywords in the literature and performed visual processing. From the perspective of high-frequency keywords, six popular research topics were summarized: (1) correlation between optic disc morphology development in glaucoma and age; (2) relationship between risk factors, such as elevated intraocular pressure and open-angle glaucoma; (3) association and variations in the prevalence and risk of eye diseases across different populations; (4) differentiation of ocular injuries using optical coherence tomography (OCT); (5) progression of normal-tension glaucoma; and (6) research on the diagnosis, classification, and image segmentation of DR (Figure 2).

### 3.5 Burst keywords analyses

**Figure 3:**
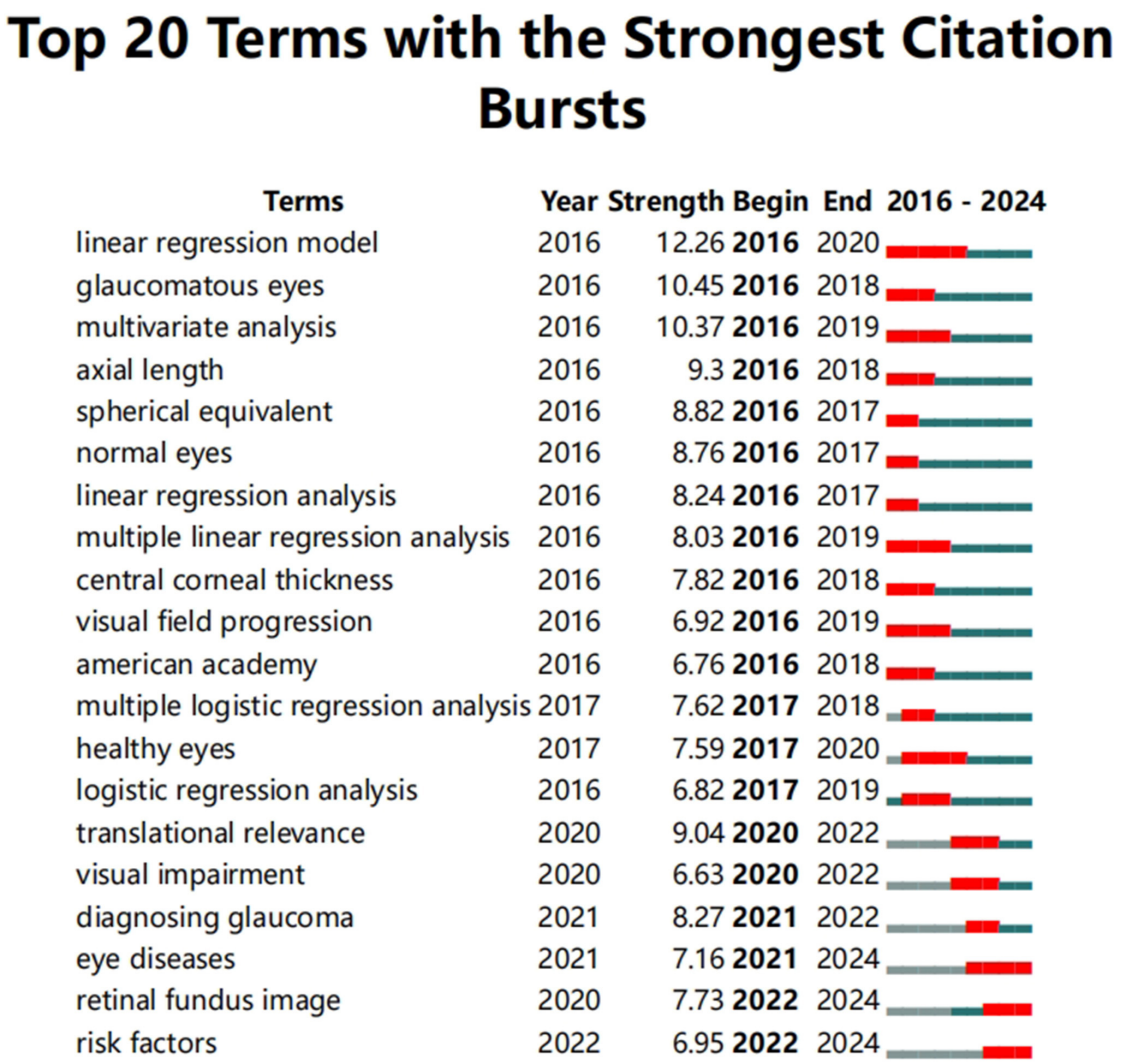
Keywords with the strongest citation bursts of publications from 2000 to 2024.

Keywords reflect current research interests, whereas burst keywords highlight emerging trends and leading edges in research. We used CiteSpace to analyze trends over time in hotspot shifts, making use of the most common keywords showing the most robust citation bursts (17). Among these were eye diseases (2021–2024), retinal fundus images (2022–2024), and risk factors (2022–2024). The most concentrated studies in these hotspots began in 2016 and continue to the present. These burst keywords emerged around 2020 or 2021 and continued to be a research hotspot until 2024. The shift in keywords reflects the change in the research focus of researchers in the field and the development process of the field. As shown in Figure 3, in the field of glaucoma research facilitated by machine learning technology, the keywords published in the literature have transitioned from terms such as linear regression model, glaucomatous eyes, multivariate analysis, axial length, spherical equivalent, and normal eyes, to eye diseases (2021–2024), retinal fundus images (2022–2024), and risk factors (2022–2024). This indicates that retinal fundus imaging technology is becoming popular, and the study of risk factors for eye diseases is becoming increasingly important (Figure 3).

## 4 Discussion

### 4.1 General data

By examining the selected articles, a total of 3,581 Science Citation Index Expanded (SCIE) papers on the application of machine learning in glaucoma were published from 2000 to 2024, demonstrating a rapid growth trend. The US contributed the largest number of articles, 1,141 (32.1 %), followed by China, 664 (18.5%). Among the top 10 leading institutions, four were based in the US and three in the UK, highlighting the significant role of researchers from these countries in advancing the field. Ophthalmology, which was the published journal most referred to (16,334 times), thereby greatly promoting the study of machine learning in glaucoma. Additionally, we looked into the 10 most-cited published articles, and the top paper was by Ting et al. (20) and published in the Journal of the American Medical Association, which had 1,369 citations. This may indicate that the paper could be a foundational or pivotal work in the field, providing essential evidence for ongoing research. These frequently acknowledged studies may provide valuable insight into this field of research.

### 4.2 The current research characteristics

The intricate and multidimensional interplay within the knowledge domain of machine learning in glaucoma warrants further examination to elucidate its conceptual structure. By analyzing the frequency and co-occurrence of high-frequency keywords, we can identify the thematic composition of research patterns and provide insights into future developments within the industry. We have summarized the research hotspots of machine learning in the field of glaucoma, which can be divided into six different perspectives.

#### 4.2.1 Correlation between the development of optic disc morphology in glaucoma and age

Machine learning technology has made significant progress in exploring the relationship between optic disc morphology and age in glaucoma. Initially, Omodaka et al. used “machine learning based on quantified eye parameters” to classify the shape of the optic disc in glaucoma, using age as one of the most important distinguishing features (21). With advancements in DL technology, researchers have discovered a positive correlation between aging and optic disc parameters, particularly an increase in the vertical cup-to-plate ratio, which is a key risk factor for glaucoma (22). By integrating machine learning with genomic data, models have identified genetic loci linked to the vertical cup-to-plate ratio, underscoring aging's pivotal role in glaucoma pathogenesis, while demonstrating robust predictive performance for glaucoma subtypes (AUCs: 0.74 for POAG, 0.73 for HTG, and 0.76 for NTG) in independent validation cohorts (23). These studies not only deepen the understanding of the pathological mechanisms of glaucoma but also provide valuable insights for early diagnosis and personalized treatment.

#### 4.2.2 Relationship between risk factors, such as elevated intraocular pressure and open-angle glaucoma

In the study of open-angle glaucoma (OAG), the application of machine learning techniques identified multiple risk factors associated with disease progression. These include the rate of optic nerve fiber layer thinning, the association between smoking and disease progression, and the association between intraocular pressure and OAG risk (24–26). Additionally, machine learning technology identified abnormal cardiac contractile potential in patients with OAG, providing a new perspective on the systemic effects of OAG (27). Moreover, machine learning demonstrated the potential for the early prediction of OAG through the analysis of electronic health records, identifying ocular hypertension as a key indicator. This highlighted the crucial role of machine learning in early diagnosis (28). Machine learning models can utilize clinical characteristics to predict the risk of ocular hypertension following silicone oil tamponade (29), achieving a highest accuracy of 0.7944 with the gradient-boosted decision trees model. These findings underscore the key role of machine learning technology in the early diagnosis and management of glaucoma.

#### 4.2.3 Association and differences in the risk and prevalence of eye diseases among different populations

Machine learning technology has significantly contributed to the diagnosis, genetic research, screening, management, and epidemiology of glaucoma. The application of DL technology has facilitated the early identification of metabolic risk factors, which may aid in the early detection of glaucoma risk (27). Machine learning has made significant advancements in automatically evaluating optic disc parameters, greatly improving diagnostic efficiency and accuracy through convolutional neural network (CNN) models. Additionally, machine learning-assisted genome-wide association studies have revealed a large number of genetic loci associated with glaucoma, offering new insights into the disease's genetic background (30). Machine learning technology has improved screening and detection capabilities for primary angle-closure glaucoma (31, 32). In patients with Parkinson's disease, machine learning technology has been utilized to assess visual dysfunction, further expanding its application in the medical field (33). These advancements not only improve research efficiency and accuracy but also provide new tools and methods for the early detection, intervention, and treatment of glaucoma.

#### 4.2.4 Differentiation of ocular injuries using OCT

The combination of machine learning techniques with OCT has revolutionized the study of the thickness assessment of the nerve fiber layer and its correlation with damage in the VF. Research has progressed from simple correlation analyses to the use of neural networks and DL algorithms to improve diagnostic accuracy (34, 35). Following the unsupervised AI for identifying the pattern of nerve fiber layer thickness associated with VF loss from OCT images, the weakly supervised DL enabled the automatic segmentation of single retinal ganglion cells in adaptive optical OCT images (36, 37). These advancements not only improve the accuracy of glaucoma diagnosis but also provide powerful tools for the early detection and personalized treatment of eye diseases.

#### 4.2.5 Progression of normal-tension glaucoma

Machine learning techniques are playing an increasingly vital role in the diagnosis, classification, progression prediction, and risk assessment of normal-tension glaucoma (NTG). With DL systems, researchers have utilized pre-trained models such as EfficientNet-b0 to achieve high accuracy (AUC = 0.98) and specificity (0.94) in NTG screening and classification (38). By integrating retinal images with clinical data, DL models have effectively predicted both the likelihood and timing of NTG conversion in normotensive glaucoma suspects, demonstrating exceptional performance (AUC = 0.994) (39). Additionally, machine learning classifiers showed superior capability in predicting NTG progression (AUC = 0.881) among young myopic patients using baseline clinical parameters alone (40). Furthermore, the combination of machine-learning algorithms with OCT angiography has provided a new perspective for NTG diagnosis and severity classification (41). A prospective cohort study on retinal vascular caliber, utilizing DL analysis, has provided a novel approach to assess factors associated with NTG progression risk (42).

#### 4.2.6 Research on the diagnosis, classification, and image segmentation of DR

The integration of DL and image processing technologies significantly improves the accuracy and efficiency of DR diagnosis. For example, a model combining pre-processing techniques and CNN demonstrated excellent performance in identifying DR, achieving an accuracy of up to 98.56% (43, 44). Further research on the depth of the preliminary training CNN model, such as EfficientNet-B7, has enabled effective multi-class classification of DR, achieving an accuracy of over 96% (45). Additionally, multi-scale residual attention networks have outperformed traditional models in retinal vascular segmentation, improving segmentation accuracy (46). Feature fusion technology, integrating wavelet packet transforms and intensity-hue-saturation features, has used a Support Vector Machine for classification to further enhance the classification performance for eye diseases (47). These studies not only promote the early diagnosis of the DR but also provide new perspectives and methods for treatment.

### 4.3 Hotspots and frontiers in research

Keywords focus on contemporary research concepts or issues while representing emerging trends and the leading edge of research. This shift in keywords reflects the evolution of the research topics. In this study, we identified hot keywords that cover the research frontiers of the subject of interest. These include eye diseases (2021–2024), retinal fundus images (2022–2024), and risk factors (2022–2024).

#### 4.3.1 Eye diseases (2021–2024)

The application of machine learning in the field of eye disease diagnosis is gradually revolutionizing traditional medical methods, not only improving the speed and accuracy of diagnosis but also providing new perspectives on disease management and treatment.

DL techniques, particularly CNN, have demonstrated high efficiency in identifying eye diseases, such as glaucoma and DR, by analyzing medical images (48). Furthermore, by integrating multimodal data, including retinal images with OCT, machine learning can provide a more comprehensive analysis of the disease and enhance diagnostic accuracy (49).

The advantages of machine learning technology are also reflected in the processing of unbalanced datasets. By modifying the loss functions, the machine learning model can effectively improve eye disease detection performance, even in unbalanced data or in the presence of outliers (50). The CNN model optimized using the pollen optimization algorithm has improved the accuracy of diagnosing diseases, such as glaucoma and cataracts in the classification of eye diseases, thereby facilitating the early detection of ophthalmic conditions (51). Furthermore, its combination with a long short-term memory network has effectively reduced errors in functional VF tests (52).

In the context of explaining the database used for training and the automated training of machine learning models, the use of generative adversarial networks for the synthesis of fundus images can meet the current limitations of the shortage of medical images and the high costs of image annotation for the rapid training of relevant machine learning diagnostic models. Integrating this with the transparency and systematization of machine learning diagnostic attention mechanisms can enhance the credibility of machine learning diagnostics, particularly in the field of ophthalmic imaging and diagnostics, where these advancements have broad applications (53). Another key application of machine learning is predicting disease progression and evaluating treatment effects, enabling personalized medical recommendations to patients. The DL model utilizes segmented OCT images to extract parameters for predicting functional VF test values. The integration of multimodal inputs can predict the progression of glaucoma. A machine learning model can also simulate glaucoma progression based on historical patient data, revealing the disease development patterns in different patients (52).

#### 4.3.2 Retinal fundus image (2022–2024)

The value of retinal fundus imaging as a core tool in the diagnosis of eye diseases has been significantly enhanced by DL technology. DL models, particularly CNNs, have been widely applied to the automated analysis of retinal fundus images, thereby improving the detection and diagnosis of major retinal diseases, such as glaucoma and DR. For example, a study integrating CNN with a visual converter significantly improved the performance of the classification of retinopathy (54). Additionally, using transfer learning and fine-tuning techniques, these models can be further optimized for specific retinal fundus image classification tasks after pre-training on public datasets such as ImageNet, achieving superior diagnostic accuracy.

Innovations in DL models continue to advance. For example, an attention-based fully CNN has been proposed for the precise segmentation of retinal blood vessels (55). This demonstrates that DL techniques can not only accelerate diagnosis but also enhance the accuracy of diagnosis by focusing on key regions within the image. Some studies have focused on image enhancement and pre-processing techniques to improve the quality of retinal fundus images, further improving model performance.

Despite the significant potential of DL in the diagnosis of eye diseases, researchers have highlighted several challenges that must be addressed. The diversity and representativeness of datasets, the ability to generalize models, and the requirement for clinical validation are current research priorities. For instance, one study combined 22 publicly available datasets to address the issue of unbalanced classes and improve the medical effectiveness of a model (56). Additionally, methods of multimodal data fusion are also being explored, integrating retinal fundus images with other imaging modalities, such as OCT, to provide a more comprehensive assessment of the disease.

#### 4.3.3 Risk factors (2022–2024)

Machine learning is playing an increasingly vital role in glaucoma risk factor analysis, early diagnosis, disease progression prediction, and patient adherence to treatment. Machine learning models are of great significance in the field of disease risk alerts, owing to their outstanding data processing and image recognition capabilities.

Through DL and multimodal recognition, machine learning can process complex medical data and identify key risk factors, such as video disc images (57). Furthermore, machine learning models can analyze surgical data to predict surgical outcomes, aiding clinical decision-making (5, 58).

The predictive power of machine learning is particularly prominent in the early detection and monitoring of glaucoma. For example, the DL model automatically evaluates the ultrasound images of biological microscopes, identifies relevant biological risk factors, and assists in the early detection of glaucoma (59). Simultaneously, by analyzing clinical records through natural language processing, machine learning can detect disease progression and predict the requirement for surgical intervention, thereby avoiding surgical risks (60).

To improve patient treatment adherence, machine learning analyzes individual differences and provides customized treatment recommendations to physicians, thereby increasing the likelihood of patients following their prescribed treatment plans (61).

The application of machine learning in glaucoma not only enhances the understanding of risk factors for glaucoma but also optimizes the diagnostic process and provides more precise medical interventions for patients.

### 4.4 Future perspectives

The integration of multimodal technology into glaucoma research offers significant potential for personalized treatment and enhanced patient outcomes. While single-mode OCT data already enables high-precision diagnosis, the combination of multimodal data may further enhance model generalization (15). Li et al. developed a multimodal DL framework that combines clinical data, VF measurements, and OCT images of 86 patients with glaucoma. This framework prognosticated glaucoma progression in VF at 12 months with an optimal area under the curve of 0.83 (62). Notably, the integration of multimodal images enabled the model to forecast vision loss with greater precision and at earlier onset. Another study introduced a new multimodal neural network designed to create a comprehensive dataset for glaucoma diagnosis and classification. To address the shortage of training data, transfer learning has also been integrated to overcome data scarcity, enabling a more effective representation of high-dimensional glaucoma-related information and enhancing model performance in multimodal analysis (52). Future studies need to further integrate multi-modal data and expand sample diversity to improve the generalization ability of the model. Bibliometric research also shows that interdisciplinary collaboration, such as computer engineering and ophthalmology, is the key to promoting the implementation of AI diagnostic systems (63).

The application of large language models (LLMs) in glaucoma management has progressed significantly, enabling rapid clinical note analysis and the extraction of critical data, such as family history, which is essential for predicting the requirement for surgical intervention. Studies indicate that LLMs achieved over 80% accuracy in predicting the probability of surgery within 1 year, thereby aiding targeted therapeutic planning to preserve visual acuity. While not infallible, they have demonstrated greater precision than traditional assessment methods in certain cases (64). A recent cross-sectional study comparing the diagnostic efficacy of an LLM chatbot (Generative Pre-trained Transformer-4) with that of glaucoma and retinal specialists has demonstrated the superior performance of LLMs in medical accuracy and response comprehensiveness, as evaluated using a Likert scale with statistical validation. These findings indicated that LLMs have the potential to enhance ophthalmological diagnostics and be integrated into clinical practice, provided they undergo further refinement and extensive patient testing. The ongoing development and validation of LLMs are essential to ensure their reliability and effectiveness in real-world settings, ultimately aiming to improve glaucoma patient care and outcomes (65).

Vision transformers (ViTs) are emerging as a breakthrough in glaucoma diagnosis, addressing challenges such as data scarcity and subtle feature discrimination. Tohye et al. propose CA-ViTs, integrating Conditional Variational GAN (CVGAN) for data augmentation and contour-guided optic disc/cup extraction, achieving 93% accuracy in multi-class classification on the SMDC dataset. This framework outperforms CNNs and vanilla ViTs (*p* < 0.01), demonstrating ViTs' potential for precise severity grading (66). Future studies include leveraging diffusion models and multi-modal data fusion to enhance diagnostic robustness.

### 4.5 Limitation

This study has some limitations. The reliance on SCIE-indexed publications may exclude relevant studies from non-indexed regional journals or preprints. Future studies should incorporate a broader range of databases, including non-English and regional sources, to provide a more comprehensive perspective on global research trends. AI, genetics, and personalized medicine could refine predictive models and treatment strategies.

This study acknowledges certain limitations in machine learning applications for glaucoma. First, the interpretability of complex models, such as deep learning, remains challenging, potentially hindering clinical trust. Second, the current generalization of machine learning models in glaucoma research is still limited by data standardization issues, such as color and resolution differences between different imaging devices (67). Third, biases in training data, such as fundus photography studies, mainly focus on specific populations, so it is especially important to be avoided (68). Addressing these limitations requires future efforts in model transparency, diverse dataset curation, and rigorous cross-validation.

## 5 Conclusion

A comprehensive, systematic, and impartial study was conducted on articles published on the application of machine learning in glaucoma research from 2000 to 1 April 2024, along with a scope review. Notably, there has been a significant surge in scholarly output post-2016, with the United States, China, England, and South Korea emerging as leading contributors to this evolving field. The thematic categorization of research has divided the multifaceted focus into six pivotal areas, with “Eye diseases”, “Retinal fundus image,” and “Risk factors” emerging as the developing directions in this field. The study's findings highlighted the broad prospects of machine learning in improving the diagnosis and treatment of glaucoma, emphasizing the requirement for ongoing research, interdisciplinary integration, and cross-disciplinary collaboration to enhance patient care and prioritize the prevention of risk factors.

## Data Availability

The original contributions presented in the study are included in the article/Supplementary material, further inquiries can be directed to the corresponding author.
